# Cancer cells arise from bacteria

**DOI:** 10.1186/s12935-018-0699-4

**Published:** 2018-12-14

**Authors:** Qing-lin Dong, Xiang-ying Xing

**Affiliations:** Department of Bioengineering, Hebei University of Technology, Tianjin, 300130 China

**Keywords:** Bacteria, Cancer cells, Origin, Cellular senescence, DNA acquisition, Hybridization, Organelle biogenesis

## Abstract

**Background:**

The origin of cancer cells is the most fundamental yet unresolved problem in cancer research. Cancer cells are thought to be transformed from the normal cells. However, recent studies reveal that the primary cancer cells (PCCs) for cancer initiation and secondary cancer cells (SCCs) for cancer progression are formed in but not transformed from the senescent normal and cancer cells, respectively. Nevertheless, the cellular mechanism of PCCs/SCCs formation is unclear. Here, based on the evidences (1) the nascent PCCs/SCCs are small and organelle-less resembling bacteria; (2) our finding that the cyanobacterium TDX16 acquires its algal host DNA and turns into a new alga TDX16-DE by de novo organelle biogenesis, and (3) PCCs/SCCs formations share striking similarities with TDX16 development and transition, we propose the bacterial origin of cancer cells (BOCC).

**Presentation of the hypothesis:**

The intracellular bacteria take up the DNAs of the senescent/necrotic normal cells/PCCs and then develop into PCCs/SCCs by hybridizing the acquired DNAs with their own ones and expressing the hybrid genomes.

**Testing the hypothesis:**

BOCC can be confirmed by testing BOCC-based predictions, such as normal cells with no intracellular bacteria can not “transform” into cancer cells in any conditions.

**Implications of the hypothesis:**

According to BOCC theory: (1) cancer cells are new single-celled eukaryotes, which is why the hallmarks of cancer are mostly the characteristics of protists; (2) genetic changes and instabilities are not the causes, but the consequences of cancer cell formation; and (3) the common role of carcinogens, infectious agents and relating factors is inducing or related to cellular senescence rather than mutations. Therefore, BOCC theory provides new rationale and direction for cancer research, prevention and therapy.

## Background

It is believed that cancer cells (PCCs) are altered normal cells formed by cell transformation. Based on this belief, extensive studies have been conducted, while the pathogenetic mechanism of cancer is, still, unclear [1]. Hence, this fundamental belief may be questionable. In the following sections, we (1) analyze the cellular processes of cancer cell formation described in the previous researches, (2) compare the similarities between cancer cell formation and cyanobacterium TDX16’s development and transition [2, 3], and (3) propose BOCC hypothesis.

## Cancer cells are “senescence-escaping cells” with unknown origin

Cancer cell formation is a complex event that can hardly be made clear in vivo. Since the landmark experiments showing that the normal cells in cultures gradually transformed into cancer cells [4, 5], in vitro cell transformation become the model experiment method to mimic in vivo cancer cell formation. The following studies manifested that in vitro cell transformation can occur spontaneously [6–9] or in the presence of physical stimuli [10], chemical carcinogens [11, 12] and viruses [13–17] in a similar pattern: the normal cells either completely failed to grow or divided for limited number of generations owing to the finite proliferative lifespan [18] and then became senescent and entered “crisis” [19], consequently most of the cells died, while only rare cells occasionally “escaped” cellular senescence or crisis and survived as the cancer cells, with altered morphology, ultrastructure, chromosomes [6, 20–22] and growth behavior properties [23, 24].

Nonetheless, the origin and developmental process of cancer cells are unknown, because cell transformations took a long period of time and the obtained cell cultures were composed of a mixture of small undifferentiated cells, polyploidy (hyperchromatic) giant cells (PGCs), flat multinucleated giant cells and sloughing, differentiating cells [7, 14, 15, 25–27], which made it difficult to track the trajectory of cancer cell development. In this case, cancer cells were postulated to be transformed from the normal cells, usually called “transformed cells”.

## PCCs/SCCs were formed in but not transformed from the senescent normal cells/PCCs

Recent studies revealed that the senescing normal cells turned into mitotically incapacitated PGCs, in which small nascent PCCs were being formed, and when PGCs burst, a large number of small PCCs were released [28–34]. Hence, PCCs are formed in but not transformed from the senescent normal cells. Similarly, new small nascent SCCs were formed in the senescent polyploid giant cancer cells (PGCCs) [32, 35–46]. These results demonstrated that PCCs and SCCs formations share a common cellular mechanism.

Nevertheless, the cellular mechanism of how the small active PCCs/SCCs were formed in the senescent unviable PGCs/PGCCs is unclear, and remains a paradox. The common explanation is that small nuclei budded from the large PGCs/PGCCs nuclei, and then became small PCCs/SCCs by re-budding off from PGCs/PGCCs, or via asymmetric cytokinesis of PGCs/PGCCs. However, indeed, nuclear budding and cytokinesis were unachievable in the senescent PGCs/PGCCs, because no energy and membrane sources were available for sustaining such active actions.

Different from the common explanation, one study showed that small SCCs were formed in the nuclei of PGCCs [35]. Similar results were frequently reported in early researches: (1) SCCs-like nuclear inclusions were developed within PGCCs nuclei [47]; and (2) PCCs-like pycnotic cells and nuclear inclusions [48], intranuclear inclusion bodies [27, 49], intranuclear membrane-bound inclusions [23] and “micronuclei” [50] were formed in PGCs nuclei, which increased in number and distributed throughout PGCs after the large nuclei disappeared. As to how the intranuclear PCCs/SCCs were formed, it was speculated that SCCs were probably derived from the ‘coiled bodies’ [35], which were, however, delimited by membranes and thus also SCCs but not the membrane-less coiled bodies [51]. Thus, there must be other precursors from which PCCs/SCCs were formed.

Since the small nascent PCCs/SCCs lack organelles and bear resemblances to the intracellular bacteria [52–61], it is likely that the small PCCs/SCCs were developed from the bacteria within PGCs/PGCCs. This notion may be intuitively unacceptable, but actually is possible in theory that eukaryotes originate from prokaryotes and supported by our finding that the cyanobacterium TDX16 (prokaryote) turned into a new alga TDX16-DE (eukaryote) [2, 3].

## PCCs/SCCs formation are similar to TDX16 development and transition

**Fig. 1:**
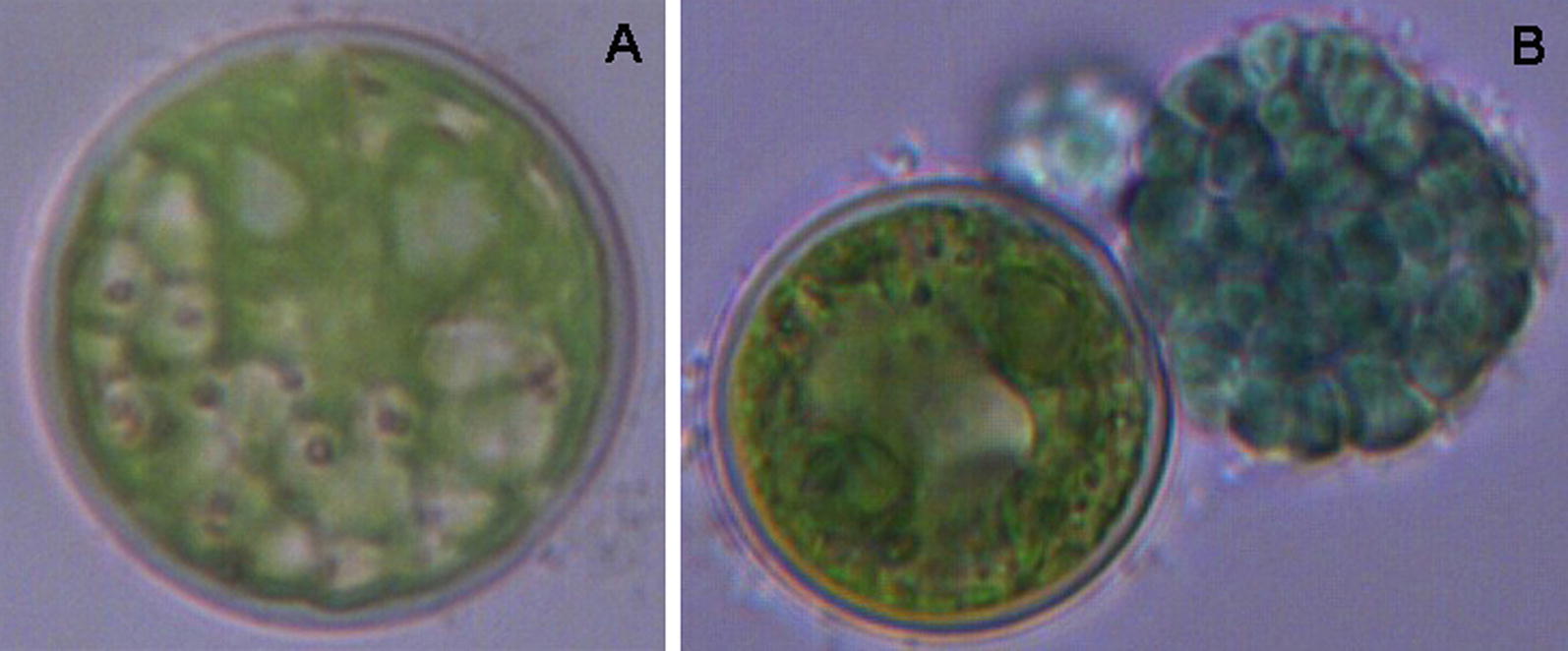
Release of TDX16 from the senescent *H. pluvialis* cell. **A** An enlarged senescent *H. pluvialis* cell. **B** A massive blue spheroid (top right) with compacted TDX16 cells was released from the ruptured senescent *H. pluvialis* cell

**Fig. 2:**
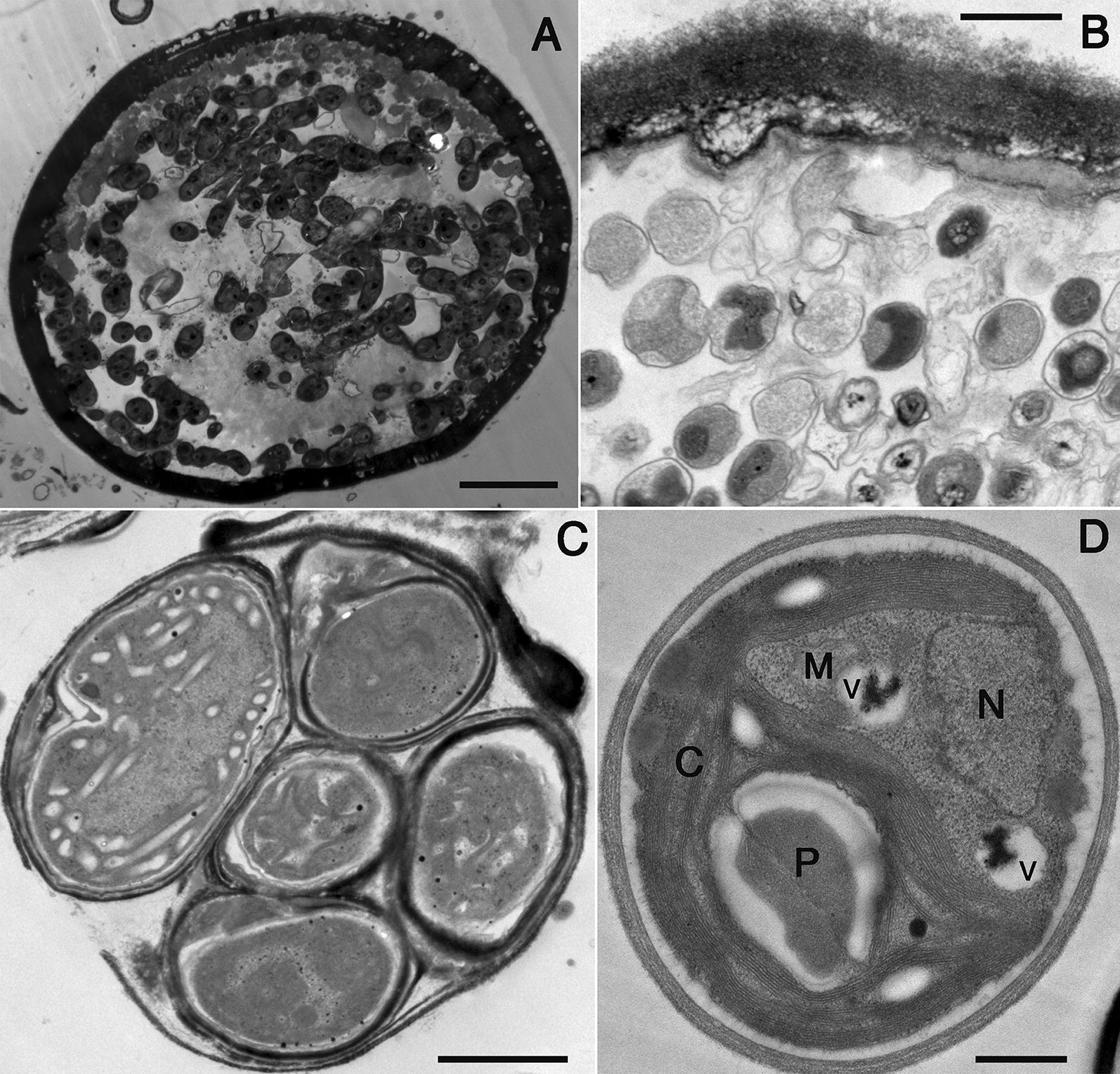
TDX16 development and transition into TDX16-DE. **A** Very small TDX16 cells with electron-dense HGBs multiplied by asymmetric division in the senescent *H. pluvialis* cell, scale bar 5 μm. **B** Small DTX16 cells filled up the cellular space of a senescent *H. pluvialis* cell, scale bar 0.5 μm. **C** Five TDX16 cells within a sporangium, scale bar 1 μm. **D** A TDX16-DE cell contains a large “e-shaped” chloroplast (C) with an embedded pyrenoid (P), a nucleus (N), a mitochondrion (M) and two vacuoles (V), scale bar 0.5 μm

*Haematococcus pluvialis* is a unicellular green alga, which grows well and synthesizes astaxanthin in the conditions of high irradiance and low temperature [62, 63]. Conversely, when cultivated under the adverse conditions of low irradiance and high temperature, the growth of *H. pluvialis* was inhibited [64]. Light microscopic observation showed that the enlarged *H. pluvialis* cells were undergoing senescence with no astaxanthin accumulation but chlorophyll reduction (Fig. 1A) and ultimately necrosis: the bloated senescent cell ruptured and liberated a massive blue spheroid consisting of countless small cyanobacterial cells (TDX16) (Fig. 1B). Transmission electron microscope observation revealed that very small premature TDX16 cells with electron dense dot-like heterogenous globular body (HGB) [3] multiplied by asymmetric division within the senescent/necrotic *H. pluvialis* cell at the expense of the dissolved organelles and cytoplasm (Fig. 2A), and subsequently enlarged into small thylakoid-less TDX16 filling up the cellular space (Fig. 2B) [64]. The liberated TDX16 was light-sensitive and unstable, which changed slowly, maintained prokaryotic state, and displayed different statuses even in the same sporangium in the dim light (Fig. 2C), but turned readily and quickly into a small green alga (TDX16-DE) by de novo organelle biogenesis as light intensity elevated (Fig. 2D) [2, 3]. TDX16-DE is a new species of green alga, containing only a double-envelope-bounded nucleus, a chloroplast, one or more mitochondria and double-membrane-bounded vacuoles, but no other organelles (Fig. 2D) [3]. Sequencing results of 16S rRNA (GenBank KJ599678.2) and Genome (GenBank NDGV00000000) indicate that TDX16 is a cyanobacterium resembling *Chroococcidiopsis thermalis,* which had acquired 9,017,401 bp DNAs with 10,301 genes from its host *H. pluvialis*. Thus, the reason for TDX16-to-TDX16-DE transition is the hybridization of the obtained eukaryotic DNA and TDX16’s prokaryotic ones, and expression of the hybrid genome [3].

TDX16’s transition demonstrates that a prokaryotic cyanobacterium can obtain its senescent algal host’s DNA and develop into a new eukaryotic alga. Since bacteria and cyanobacteria are close relatives sharing similar structures and behaviors, it is possible that some bacteria, like TDX16, are capable of prokaryote-to-eukaryote transition under the similar conditions. If so, the bacteria within normal/cancer cells of multicellular eukaryotes may develop into new single-celled eukaryotes: PCCs/SCCs. Consistent with this notion, PCCs/SCCs formation really share striking similarities with TDX16 development and TDX16-to-TDX16-DE transition (Figs. 1, 2):

1. Similar to TDX16 development, the nascent PCCs/SCCs (1) are formed within PGCs/PGCCs, (2) reproduce by asymmetric division, which is usually interpreted as the unachievable division of PGCs/PGCCs or subnuclei, (3) aggregate into spheroids [28, 31, 33, 39, 41–46], and (4) are released after PGCs/PGCCs burst [28, 41].
2. The newly released/formed PCCs/SCCs are very small and undifferentiated [15, 35, 46, 65, 66] containing only “micronuclei” [27, 35], which are, however, not the real nuclei but the single-membrane-bounded DNA storage bodies (DSBs), similar to HGBs in TDX16 (Fig. 2A, B) [3]. So, just as TDX16, PCCs/SCCs are initially absent of organelles.
3. Although how and when organelles are developed in the small PCCs/SCCs remain unknown, it is certain that with the enlargement of latter organelles are formed de novo in a way akin to organelle biogenesis in TDX16 that resulted in its transition into eukaryotic TDX16-DE (Fig. 2D) [3].

## Presentation of the hypothesis

**Fig. 3:**
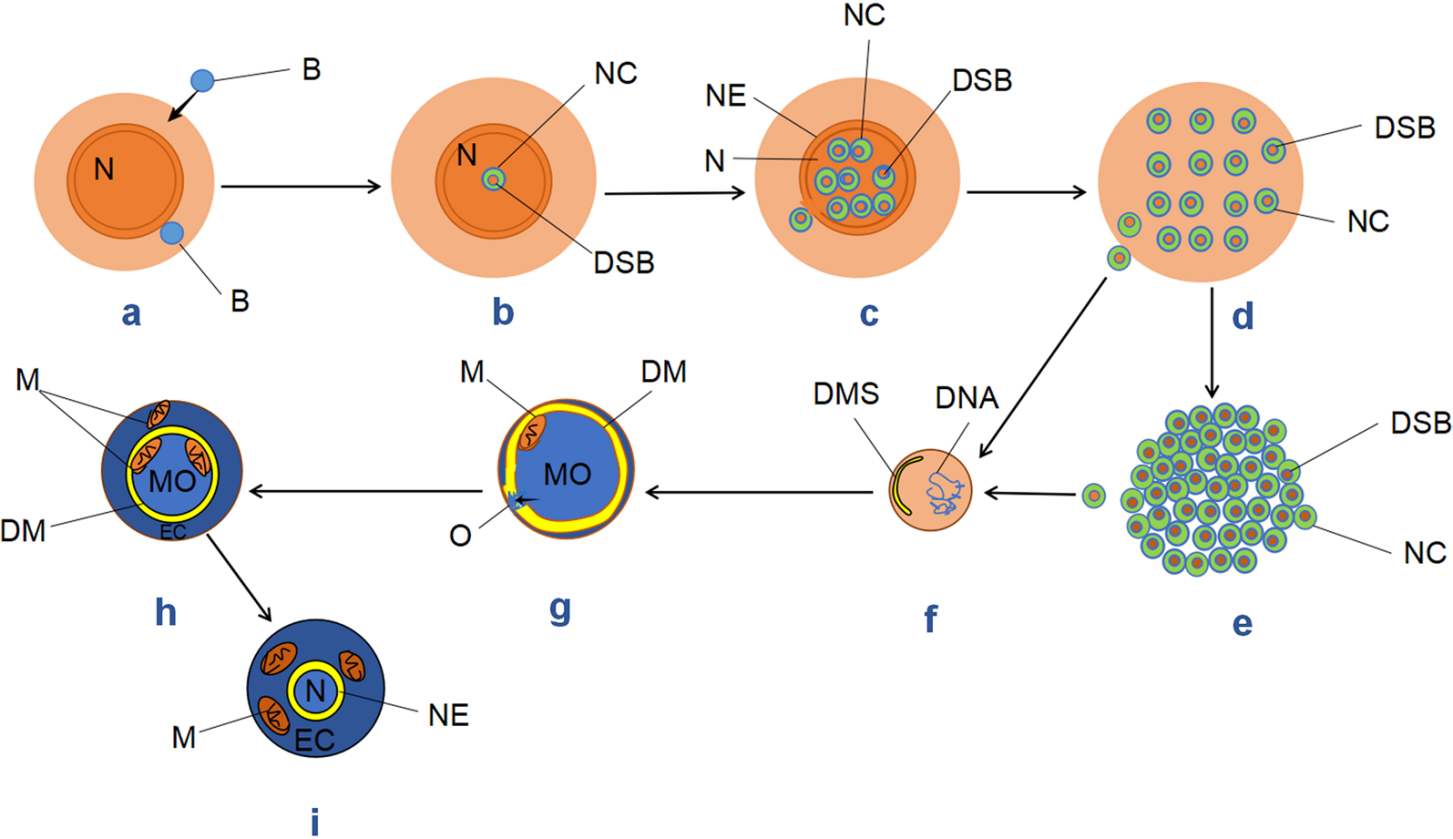
Cartoon illustrating the formation of cancer cells. **a** The normal cell/PCC undergoes senescence and bloats into a PGC/PGCC with a large nucleus (N). leading to the activation of the dormant intracellular bacterium (B) and the invasion of the extracellular bacterium. **b** The bacterium intrudes into the large PGC/PGCC nucleus, takes up the nuclear DNA and retains the obtained DNA in DSB, and thus turns into the small nascent cancer cell (NC): PCC/SCC. **c** NC multiplies in the nucleus by asymmetric division, some of which penetrate the nuclear envelope (NE) into the cytoplasm. **d** All NCs enter cytoplasm after the rupture of NE, and continue to proliferate. During this process, some NCs protrude and escape from the necrotic PGCs/PGCCs; while most NCs aggregate into a spheroid. **e** A spheroid consist of NCs is liberated from the ruptured PGCs/PGCCs. **f** As NC increases in size, DSB disrupts and thus the acquired eukaryotic DNA and the bacterial one fragments and hybridizes into a hybrid genome; concurrently, a peripheral double membrane segment (DMS) is synthesized by fusion of the cytoplasmic membrane-derived vesicles. **g** NC further enlarges, DMS extends into a closed double membrane (DM), enclosing the total cytoplasm, and thus gives rise to a single multifunctional organelle (MO). Inside MO, a mitochondrion (M) is assembled by encapsulating the selected relevant components (e.g., DNA) with the double membrane synthesized by fusion of the inner DM membrane-derived vesicles; meanwhile, a small opening (O) is formed on DM allowing the selective release of MO matrix. **h** The released MO matrix builds up the eukaryotic cytoplasm (EC), and the newly assembled mitochondrion detaches from MO into EC; while new mitochondria continue to develop in MO, resulting the diminishment of MO. **i** After all mitochondria enter EC, MO dwindles into a nucleus (N), such that NC develops into a large mature PCC/SCC

Based on the above analyses and comparisons, we propose the bacterial origin of cancer cells (BOCC) that PCCs/SCCs arise from bacteria through 8 steps (Fig. 3):

1. Under the stress of physical, chemical or biological factors, normal cells/PCCs undergo senescence, turn into PGCs/PGCCs with large nuclei and lose the ability to control the intracellular bacteria and keep out the extracellular ones, leading to activation of the former and invasion of the latter (Fig. 3a).
2. The intracellular bacteria usually reside in PGCs/PGCCs cytoplasm. Occasionally, one or several bacteria intrude into the large PGCs/PGCCs nuclei, take up the active nuclear DNAs (including related proteins) and retain the acquired DNAs in the replicable membrane-delimited DSBs. As such, the DSB-containing bacteria in PGCs/PGCCs turn into the small nascent cancer cells (NCs): PCCs/SCCs (Fig. 3b), which are usually mistaken for “small nuclei” or “micronuclei” in PGCs/PGCCs and “multinucleated giant cells”/“multinucleated giant cancer cells”.
3. The small NCs reproduce via asymmetric division in PGCs/PGCCs nuclei, some of which penetrate the nuclear envelope (NE) into the cytoplasm (Fig. 3c).
4. All NCs enter cytoplasm after the rupture of NE, and continue to proliferate at the expense of degraded organelles and cytoplasm. During this process, some NC protrude and escape from the necrotic PGCs/PGCCs (Fig. 3d); while most NCs aggregate into a spheroid, which is liberated after the burst of PGCs/PGCCs (Fig. 3e).
5. As NC increases in size, DSB disrupts and thus the acquired eukaryotic DNAs and the bacterial DNAs fragment and hybridize into a hybrid genome in a way similar to the genome assembly of TDX16-DE [3] and “chromothripsis” [67]; concurrently, a peripheral double membrane segment (DMS) is synthesized by fusion of the cytoplasmic membrane-derived vesicles (Fig. 3f).
6. NC further enlarges, DMS extends into a closed double membrane (DM) enclosing the total cytoplasm, and thus gives rise to a single multifunctional organelle (MO). Inside MO, mitochondria are assembled by encapsulating the relevant components (e.g., DNAs) with the double membranes synthesized by fusing the inner DM membrane-derived vesicles; meanwhile, a small opening (O) is formed on DM allowing the selective release of MO matrix (Fig. 3g).
7. The released MO matrix builds up the eukaryotic cytoplasm (EC), and the newly assembled mitochondria detach from MO into EC; while new mitochondria are developed continuously in MO, resulting in the diminishment of MO (Fig. 3h).
8. After all mitochondria enter EC, MO dwindles into a nucleus (N), such that NCs develop into large mature PCCs/SCCs (Fig. 3i).

In the way described above, new SCCs can be developed successively from the same or different species of bacteria, which inevitably result in the increased heterogeneity, genetic instability and endless complexity of cancer cells in vivo and in vitro. Hence, PCCs, SCCs and the subsequent new SCCs look superficially like the putative “cancer stem cells” [68] in cancer progression.

## Evidences supporting the BOCC hypothesis

1. There are 10× more bacterial cells than human cells in the human body [69].
2. Bacteria play a key role in carcinogenesis. *Helicobacter pylori* is a definite carcinogen for gastric cancer [70]. Many other bacteria are related with various cancers, such as *Salmonella typhi* [71], *Chlamydia pneumoniae* [72], *Mycoplasma hominis* [73], *Bacteroides fragilis* [74], *Streptococcus bovis* [75], *Escherichia coli* [76], *Fusobacterium* spp. [77]. and *Neisseria gonorrhoeae* [78].
3. Like bacteria and single-celled eukaryotes (protists, e.g., yeast), cancer cells can grow in agar medium and form colonies [79], proliferate in the absence of anchorage in vitro [80] and ferment glucose in the absence of oxygen (anaerobic fermentation) with the production of lactic acid [81].
4. Cancer cell genomes were assembled from DNA fragments all at once in a single catastrophic event (chromothripsis) [67].
5. Cancer cell genomes contain bacterial DNA [82–84].
6. Genes of ancient and unicellular origin are highly and preferentially expressed during tumorigenesis [85, 86].

## Testing the hypothesis

The following BOCC-based predictions can be used for testing the hypothesis:

1. No bacteria, no cancer. So, after eliminating the intracellular bacteria, normal cells can not “transform” into cancer cells in any conditions.
  *Testing* Inducing the bacteria-containing and bacteria-free normal cells with different carcinogens and infectious agents and tracking their changes.
2. The “small nuclei” in “multinucleated giant cells”/“multinucleated giant cancer cells” and PGCs/PGCCs as well as the chromothripsis-related “micronuclei” [87–89] are the bacteria-derived nascent PCCs/SCCs, containing bacterial and host’s DNAs.
  *Testing* Isolating the “small nuclei” and “micronuclei”, sequencing their DNAs and identifying bacterial DNAs.
3. The “small nuclei” and “micronuclei” mentioned above have no organelles but DSBs, which enlarge and develop into eukaryotic cancer cells by de novo organelle biogenesis after disruption of DSBs.
  *Testing* Cultivating the isolated “small nuclei” and “micronuclei” and track microscopically the changes of cellular structure.
4. There is an outer membrane external to the cytoplasmic membrane of “small nuclei”, “micronuclei” and PCCs/SCCs (except those that arose from the cell wall-less bacteria e.g., *Mycoplasma*), while the cytoplasmic membrane is capable of oxidative phosphorylation and lipid synthesis.
  *Testing* Detecting the outer membrane, and identifying the electron carriers of oxidative phosphorylation and the relevant enzymes in the cytoplasmic membrane.
5. PCCs/SCCs genomes contain massive hybrid genes with bacterial gene sequences.
  *Testing* Identification of bacterial DNA sequences in PCCs/SCCs genomes.

## Implications of the hypothesis

### Clarifying the cause and results of cancer

Cancer cell genomes are formed by hybridizing the acquired eukaryotic DNAs and bacterial ones. Such hybridization inevitably result in (1) gene loses, substitution and synthesis, which are usually ascribed to “mutations” (DNA changes), and (2) aneuploidy and karyotypic heterogeneity, which are considered to be the reasons for genome instability. Therefore, genetic changes and instabilities are not the causes, but the consequences of cancer cell development.

### Explaining the reason behind the hallmarks of cancer

In the light of BOCC the reason behind the hallmarks of cancer [90, 91] become clear. Cancer cells are new bacteria-derived single-celled eukaryotes formed by expression of the hybrid genomes, which inevitably exhibit the characteristics of protists, e.g., “self-sufficiency”, “replicative immortality”, “invasion and metastasis” and somatic cells, i.e. “angiogenesis”, but evade somatic-cell-targeted controls of “growth suppress” and “apoptosis”. Indeed, “reprogramming energy metabolism” that cancer cells generate energy via ‘‘aerobic glycolysis”, i.e. “Warburg effect” [81], is also a characteristic of protists, because the yeast *Saccharomyces cerevisiae* produce energy via aerobic fermentation [92] called “Crabtree effect” [93]. As to “evading immune destruction”, the reasons may be that the cancer-causing bacteria have already developed strategies to evade immune system defenses as indicated by their persistence within the host cells, so the bacteria-derived cancer cells acquire the capability to evade immune destruction.

### Clarifying the common role of carcinogens, infectious agents and relating factors in carcinogenesis

Cancer can be caused by physical and chemical carcinogens [94], infectious agents [95] including viruses [96], bacteria [70] and parasites [97], and related to the factors of organismal aging [98], immunity [99] and inflammation [100]. It is reasonable that these varied carcinogens, infectious agents and relating factors play or link to a common role in carcinogenesis. Such a common role is unknown, but apparently not mutation, because most of the carcinogens and infectious agents are not mutagenic.

According to BOCC theory, senescence of normal cells is the prerequisite for the formation of bacteria-derived cancer cells. Hence, the common role of carcinogens, infectious agents and relating factors in carcinogenesis is inducing or linked to cellular senescence. In general, carcinogens and infectious agents induce cell senescence by (1) causing cell damage and or more or less DNA changes (physical and chemical carcinogens), (2) integrate DNA into host cell genome to interfere cell metabolic activities (viruses), (3) mechanical irritation (parasites), (4) secreting irritant metabolites (bacteria and parasites), (5) inducing chronic inflammation (physical and chemical carcinogens, bacteria, viruses, and parasites) and (6) interfering or inhibiting host’s immune system resulting in lowered immunity or immunosuppression (physical and chemical carcinogens, bacteria, viruses, and parasites); while organismal aging is associated with immunity declines and thus cellular senescence.

### Providing a new rationale and direction for cancer research, prevention and therapy

If confirmed, BOCC theory provides a new rationale for cancer research, and a new direction for cancer prevention and therapy. Eliminating the cancer-causing bacteria in the body with antibiotics or other agents, stopping bacteria’s acquisition and hybridization of host’s DNAs, and developing bacteria-targeted vaccines can prevent cancer and inhibit, to some extent, cancer progression. Identifying the differences between the bacteria-derived cancer cells and normal cells in structure, constitution and metabolism can provide targets for developing drugs and vaccines to eradicate cancer cells in cancer treatment.

## Abbreviations

B: bacteria
BOCC: bacterial origin of cancer cells
DSBs: DNA storage bodies
DM: double membrane
DMS: double membrane segment
EC: eukaryotic cytoplasm
HGB: heterogenous globular body
M: mitochondria
MO: multifunctional organelle
N: nucleus
NC: nascent cancer cells
NE: nuclear envelope
O: opening
PCCs: primary cancer cells
PGCs: polyploidy giant cells
PGCCs: polyploid giant cancer cells
SCCs: secondary cancer cells
